# First epidemiological survey of *Toxoplasma gondii* in Galapagos sea lions (*Zalophus wollebaeki*)

**DOI:** 10.1051/parasite/2025028

**Published:** 2025-06-11

**Authors:** Juan D. Mosquera, Eduardo Diaz, Rosa de los Ángeles Bayas, Diego Páez-Rosas, Colón Jaime Grijalva-Rosero, Sonia Zapata, Sandie Escotte-Binet, Quentin Di Brasi, Isabelle Villena, Marie-Lazarine Poulle

**Affiliations:** 1 UR ESCAPE, University of Reims Champagne-Ardenne (URCA) 51095 Reims France; 2 Instituto de Microbiología, Universidad San Francisco de Quito (USFQ) 170901 Quito Ecuador; 3 Colegio de Ciencias Biológicas y Ambientales COCIBA, Universidad San Francisco de Quito (USFQ) 170901 Quito Ecuador; 4 Escuela de Medicina Veterinaria, Colegio de Ciencias de la Salud, Universidad San Francisco de Quito (USFQ) 170901 Quito Ecuador; 5 Fundación GAIAS Europa de la Comunitat Valenciana (GAIAS) 46003 Valencia España; 6 Galapagos Science Center (GSC), USFQ & UNC-Chapel Hill 200101 Isla San Cristóbal Galápagos Ecuador; 7 Fundación Conservando Galápagos, Galapagos Conservancy 200102 Isla Santa Cruz Galápagos Ecuador; 8 Dirección Parque Nacional Galápagos, Oficina Técnica San Cristóbal 200101 Isla San Cristóbal Galápagos Ecuador; 9 Parasitology-Mycology Service, National Reference Center (CNR) for Toxoplasmosis, Biological Resource Center (CRB) Toxoplasma, University Hospital Center (CHU) of Reims 51092 Reims France

**Keywords:** Marine mammals, Toxoplasmosis, Zoonotic diseases, Environmental contamination

## Abstract

*Toxoplasma gondii* is the protozoan parasite responsible for toxoplasmosis, a zoonosis that represents a health risk for mammals, including marine species. Felines are the only definitive hosts of this parasite, playing a critical role in the introduction and maintenance of the pathogen in a new environment. Recent data demonstrate the contamination by *T. gondii* of the terrestrial and seawater environment of the Galapagos archipelago, in the Pacific Ocean. Little is known about the exposure of Galapagos’ threatened species to *T. gondii*, although introduced domestic cats in the archipelago are known to be seropositive for *T. gondii*. We documented for the first time exposure to *T. gondii* of Galapagos sea lions (*Zalophus wollebaeki*), an endemic and emblematic species of the archipelago. The modified agglutination test revealed the presence of antibodies against *T. gondii* in 61 of 77 plasma samples collected in 2016–2017 from 2- to 4-year-old wild sea lions live-handled in their breeding sites on the inhabited island of San Cristóbal. Antibodies were also detected in 4 of 19 serum samples (21%) from sea lions whose corpses were found in 2021 on the same island. In addition, *T. gondii* DNA was detected in a lung sample from one necropsied pup and a tissue cyst-like structure was found in another, suggesting infection. These results, together with the high prevalence of antibodies in 2 to 4-year-olds, indicate that Galapagos sea lions are frequently exposed to *T. gondii* and raise concerns that toxoplasmosis may pose a threat to this endemic species.

## Abstract (fr)

*Toxoplasma gondii* est le parasite protozoaire responsable de la toxoplasmose, une zoonose qui représente un risque sanitaire pour les mammifères, y compris les espèces marines. Les félins sont les seuls hôtes définitifs de ce parasite, jouant un rôle essentiel dans l’introduction et le maintien de l’agent pathogène dans un nouvel environnement. Des données récentes mettent en évidence la contamination par *T. gondii* de l’environnement terrestre et marin de l’archipel des Galápagos, dans l’océan Pacifique. On sait peu de choses sur l’exposition des espèces menacées des Galápagos à *T. gondii*, bien que les chats domestiques introduits dans l’archipel soient connus pour être séropositifs à *T. gondii*. Nous documentons pour la première fois l’exposition à *T. gondii* des otaries des Galápagos (*Zalophus wollebaeki*), une espèce endémique et emblématique de l’archipel. Le test d’agglutination modifié a révélé la présence d’anticorps contre *T. gondii* dans 61 des 77 échantillons de plasma prélevés en 2016–2017 sur des otaries sauvages âgées de 2 à 4 ans, manipulées vivantes dans leurs sites de reproduction sur l’île habitée de San Cristóbal. Des anticorps ont également été détectés dans 4 des 19 échantillons de sérum (21 %) provenant d’otaries dont les cadavres ont été retrouvés en 2021 sur la même île. De plus, de l’ADN de *T. gondii* a été détecté dans un échantillon de poumon d’un juvénile autopsié et une structure ressemblant à un kyste tissulaire a été trouvée chez un autre, suggérant une infection. Ces résultats, associés à la forte prévalence d’anticorps chez les otaries âgées de 2 à 4 ans, indiquent que les otaries des Galápagos sont fréquemment exposées à *T. gondii* et suscitent des inquiétudes quant à la menace que la toxoplasmose pourrait représenter pour cette espèce endémique.

## Introduction

*Toxoplasma gondii* is the causative agent of toxoplasmosis and a cosmopolitan protozoan parasite that has long been recognized for its adaptability and capacity to infect a wide range of warm-blooded hosts, including humans and various species of mammals and birds [10]. The complex life cycle of this protozoan involves felines as the definitive hosts that shed millions of oocysts into the environment through their feces [15]. Oocysts are the environmentally resistant infective forms of the parasite that can persist for several months to years in humid environments and be carried from land to coastal waters *via* runoff and rivers [20]. From here, oocysts infiltrate the marine food chain by being deposited on the surfaces of macroaggregates and seaweed that are consumed by invertebrates and fish [42, 43]. Furthermore, fish can transport oocysts serving as both vehicles of *T. gondii* dispersal and source of infection for marine mammals and birds [1, 23].

In all populated regions of the world, the domestic cat (*Felis silvestris catus*) is the main cause of environmental contamination by *T. gondii*. The introduction of cats into insular environments and the recent increase in their populations on inhabited coastlines are exacerbating the transfer of oocysts into coastal waters, raising concerns about their potential impact on seabirds and marine mammals [41]. These marine homeotherms may be particularly susceptible to acute infections since their immune systems are naïve to *T. gondii* given their limited exposure history. Along the California coast, *T. gondii* has notably been identified as a significant cause of mortality in sea otters (*Enhydra lutris*) with infections leading to fatal meningoencephalitis [17].

The presence and pathogenicity of *T. gondii* has also been reported in other marine mammals such as cetaceans and pinnipeds [11]. Specifically, in pinnipeds, exposure to *T. gondii* and disease have been described primarily in seals inhabiting the Antarctic and Canada [24, 33]. Less information is available on these infections in otariids; however, seropositivity was reported in Antarctic fur seals (*Arctocephalus gazella*) and New Zealand sea lions (*Phocarctos hookeri*) [25]. Furthermore, clinical toxoplasmosis was reported in a South American fur seal (*Arctocephalus australis*) from Brazil [34] and in 12 California sea lions (*Zalophus californianus*) along the central California Coast [2].

In the Galapagos archipelago (Ecuador), domestic cats were introduced in the 19th century and are ubiquitous on the four inhabited islands [32]. Galapagos domestic cats have shown a high seroprevalence of *T. gondii* [19]. Antibodies against *T. gondii* have also been reported in land birds, seabirds, as well as *T. gondii* DNA in oysters, suggesting the presence of oocysts in the ecosystems of this archipelago [5, 6, 27, 28]. This contamination by *T. gondii* raises questions about its potential impact on the iconic Galapagos sea lion (*Zalophus wollebaeki*), which is one of the main tourist attractions of the region [21].

The Galapagos sea lion is listed as endangered by the International Union for Conservation of Nature [45]. Its population is in decline with numbers around 20,000 individuals, distributed across all islands of the archipelago [31]. This species inhabits an ecosystem with frequent periods of low productivity (*e.g.*, El Niño event) that generate food stress and increases in mortality rates [37]. As a result, the potential infectious disease spread from domestic animals (*i.e.*, dogs and cats) has become a significant conservation issue [13, 40]. Furthermore, its status as a top predator makes it a sentinel species for the Galapagos National Park Directorate, since it plays an important role in maintaining the functional biodiversity of the marine ecosystems in the region [29].

Despite the worldwide range and broad marine host record of *T. gondii* infection, there is no evidence regarding this zoonotic pathogen in most parts of the world [1]. In this study, we examined the exposure of Galapagos sea lions to *T. gondii* in some of the most important breeding colonies for the species located on San Cristóbal Island, including the urban limits of Puerto Baquerizo Moreno, one of the most populated of the archipelago [36]. Our research aimed to determine a first insight of the species’ exposure and susceptibility to *T. gondii*. The results provide useful information to public health, veterinary medicine, and biodiversity conservation stakeholders for the development of effective prevention and control measures.

## Materials and methods

### Study area

**Figure 1:**
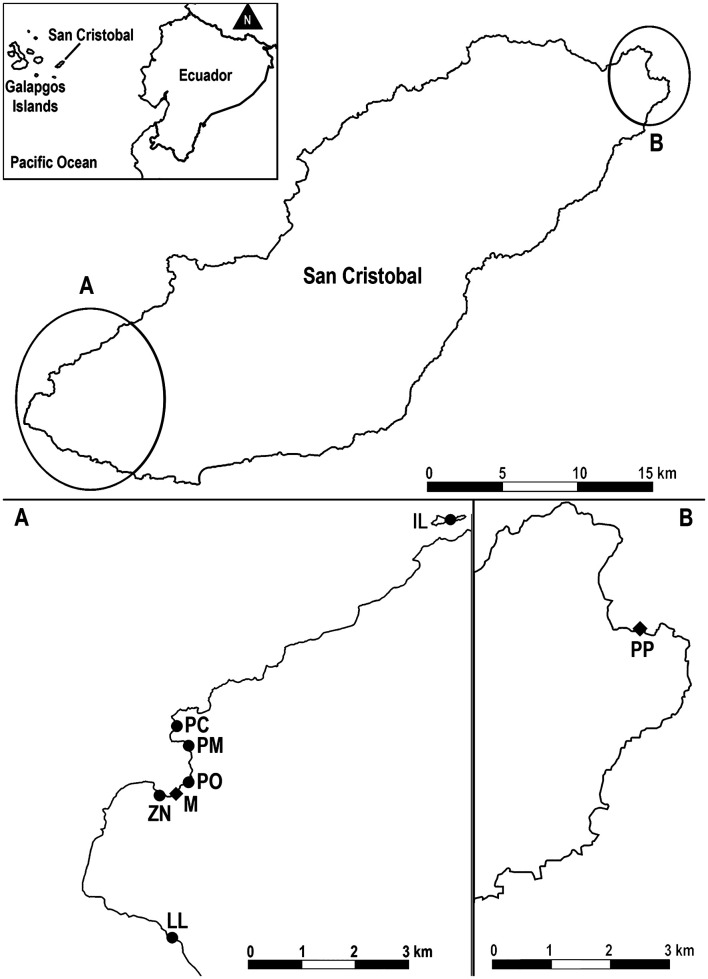
Location of the Galapagos Islands, San Cristóbal Island and of the rookeries (circles: 2021 sampling, diamonds: 2016–2017 sampling) where Galapagos sea lions were sampled. A: La Lobería (LL), Zona Naval (ZN), Malecón (M), Playa de Oro (PO), Playa Mann (PM), Punta Carola (PC), Isla Lobos (IL). B: Punta Pitt (PP).

The Galapagos archipelago is situated in the Eastern Tropical Pacific Ocean, at approximately 1000 km from continental Ecuador (Fig. 1). The climate is subtropical with a hot season from January to May and a cool season from June to December [47]. A barren landscape dominates, except at higher altitudes that receive enough rain to support a lush, tropical environment. The present study was conducted on San Cristóbal Island breeding colonies (Fig. 1), which host more than 10% of the total Galapagos sea lion population [31, 38].

### Sample collection and ethics

Two sets of samples were used in this study. The first one comprised plasma samples collected from 77 Galapagos sea lions (aged 2–4 years) live-handled between 2016 and 2017 as part of a previous health assessment conducted by Páez-Rosas *et al.*, Universidad San Francisco de Quito (USFQ). These sea lions were sampled at two locations: Malecón (*n* = 27) and Punta Pitt (*n* = 50) sites (Fig. 1). The methods for capturing, handling, and blood collection followed the protocols described by Páez-Rosas *et al.* [30] and were carried out under the supervision and approval of the Galapagos National Park Directorate (DPNG) and Ecuador’s Ministerio del Ambiente, Agua y Transición Ecológica (MAATE) and under permit Nos. PC-31-21, MAATE-DBI-CM-2021-0178, and 032-2023-EXP-CM-FAU-DBI/MAATE.

The second set of samples included organs and sera collected from sea lion carcasses during the 2021 birthing season (September–October). These samples were collected exclusively for the present study by Mosquera *et al.* (USFQ and URCA UR ESCAPE). Patrols were conducted twice daily, in the early morning (~6:00 AM) and evening (~6:00 PM), at La Lobería, Zona Naval, Playa de Oro, Playa Mann, Punta Carola and Isla Lobos rookeries (Fig. 1). The carcasses found were initially reported to the DPNG and then transported with the aid of a ranger to their installations to perform necropsies. Organ sample collection and processing followed the approved protocols of the DPNG and MAATE, under the same research permits as noted above.

The sex of the individuals was reported, and their age was estimated using the morphometric measurement criteria developed by Jeglinski *et al.* [16]. The sampled individuals were grouped into three age classes according to Riofrío-Lazo & Páez-Rosas [37] and Wolf and Trillmich [49]: pup (<1 year), juvenile (1–4 years) and adult (4+ years). When possible, blood from carcasses was collected from the heart and chest cavity, centrifuged to obtain serum and stored at −20 °C. As brain, heart and lung were previously selected for the detection of *T. gondii* in the California sea lion [2], sections of these organs from individual sea lions were collected in 50 mL Falcon tubes with 96% ethanol for molecular analyses and formalin 10% for immune-histopathological analyses. Samples in ethanol were stored at −20 °C, samples in formalin were stored at room temperature at the Galapagos Science Center (USFQ) at San Cristóbal until they were shipped to the UR ESCAPE laboratory in France.

### Serological analysis

Sera samples were examined using the modified agglutination test (MAT) for the detection of antibodies against *T. gondii* [8]. Formalized tachyzoites obtained from the National Reference Center on Toxoplasmosis (Reims, France) were used as antigen. All samples were tested at 1:10, 1:20, 1:40, 1:80, 1:160 and 1:320 dilutions. Agglutination at 1:10 or higher was considered as positive [8].

### Molecular detection

Total genomic DNA was obtained from heart, brain and lung samples of all individuals sampled in 2021 for the detection of *T. gondii*. Each sample was minced, treated with trypsin, filtered and pelleted by centrifugation using a modified protocol [14]. DNA was extracted from the pellets using a QIAamp DNA minikit (QIAGEN, Hilden, Germany), following the manufacturer’s instructions. Detection of *T. gondii* DNA was performed by TaqMan real-time qPCR [18] in a QuantStudio 5 thermocycler (Thermo Fisher).

### Histopathological and immunohistochemical examination

Budget constraints reduced the histopathological and immunohistochemical analysis to only hearts and brains, organs where a high prevalence of *T. gondii* can usually be found [9, 39]. Sections of the hearts and brain samples were embedded in paraffin and stained with hematoxylin and eosin. The slides with the stained heart and brain sections were sent to the Vet Diagnostics laboratory (Charbonnières-les-Bains, France) for histopathological screening of lesions compatible with an infection caused by a coccidian parasite. The slides for which this type of lesion was identified, accompanied by a negative control, were processed by immunohistochemistry at the Center Léon Bérard in Lyon on a Benchmark Ultra machine (Roche, Basel, Switzerland), using an UltraView Universal DAB Detection Kit (253-4291, Roche).

For immunohistochemistry analyses, 4 μm thick sections were made with a microtome (HM340E, MMF) then spread on TOMO slides (TOM-1190, VWR). These slides were deparaffinized with Ventana EZ Prep reagent (950-102, Ventana) and hydrated, followed by antigen unmasking using Ventana Tris-EDTA buffer pH 8 to 8.5 (CC1 buffer, 950-124, Roche). Anti-toxoplasma antibody (RB-9423-P0, Thermo) was diluted with Microm (F/936B-08, MMF) and incubated for 32 min. Diaminobenzidine (DAB) was used for revelation, then the joint application of hematoxylin II (8 min) and bluing (4 min) allowed for staining of the cell nuclei.

## Results

### Collection of Galapagos sea lion carcasses

The carcasses of 28 individuals (15 males and 13 females) between 0–4 years of age (*i.e.* pups and juveniles) and one adult (male) were collected during the sampling of 2021 (Table 1). The adult carcass was delivered from Playa del Oro after being hit by a car. Individual 22 was brought to the PNG facilities from Playa Mann, but died shortly after.

**Table 1:** Detection of *Toxoplasma gondii* antibodies in Galapagos sea lions of the 2021 sampling period.

Site	ID	Age class (sex)	MAT results (titer)
Isla Lobos	29	Pup (f)	NA
La Lobería	6	Pup (m)	–
	11	Pup (f)	–
	1	Pup (f)	NA
	13	Pup (f)	–
Zona Naval	4	Pup (m)	–
	5	Pup (f)	+ (1:20)
	7	Pup (f)	–
	15	Pup (m)	–
	16	Pup (f)	NA
	25	Pup (f)	NA
	26	Pup (f)	NA
Playa de Oro	10	Pup (m)	–
	24	Pup (m)	NA
	9	Pup (f)	–
	23	Adult (m)	+ (1:80)
Playa Mann	3	Pup (m)	NA
	12	Pup (m)	–
	14	Pup (f)	–
	18►	Pup (m)	–
	19	Pup (m)	–
	22	Juvenile (f)	–
Punta Carola	2	Pup (m)	NA
	8	Juvenile (m)	+ (1:60)
	17*	Pup (m)	+ (1:80)
	20	Pup (m)	-
	21	Pup (m)	-
	27	Pup (f)	NA
	28	Pup (m)	NA

NA: Not available, serum could not be collected. ►A structure resembling a tissue cyst was found in the heart of individual 18. *Toxoplasma gondii DNA was only detected in duplicate in a lung sample of individual 17 (Cq mean 39.38).

### Detection of antibodies against *Toxoplasma gondii*

Out of the 77 individuals sampled between 2016 and 2017, 61 (79.2%) presented antibodies against *T. gondii* with titers ranging from 1:10 to 1:320 (Table 2)*.* For the 2021 samples, serum was obtained from 19 out of the 29 sea lion carcasses whose blood was not yet coagulated when necropsied. Out of these 19 individuals, four had antibodies against *T. gondii* (21.05%) (Table 1).

**Table 2:** Number of MAT-positive Galapagos sea lions of the 2016–2017 sampling period.

						
MAT titer	1:10	1:20	1:40	1:80	1:160	1:320
Sample number	1	19	19	17	4	1

### Immunohistopathological analyses

**Figure 2:**
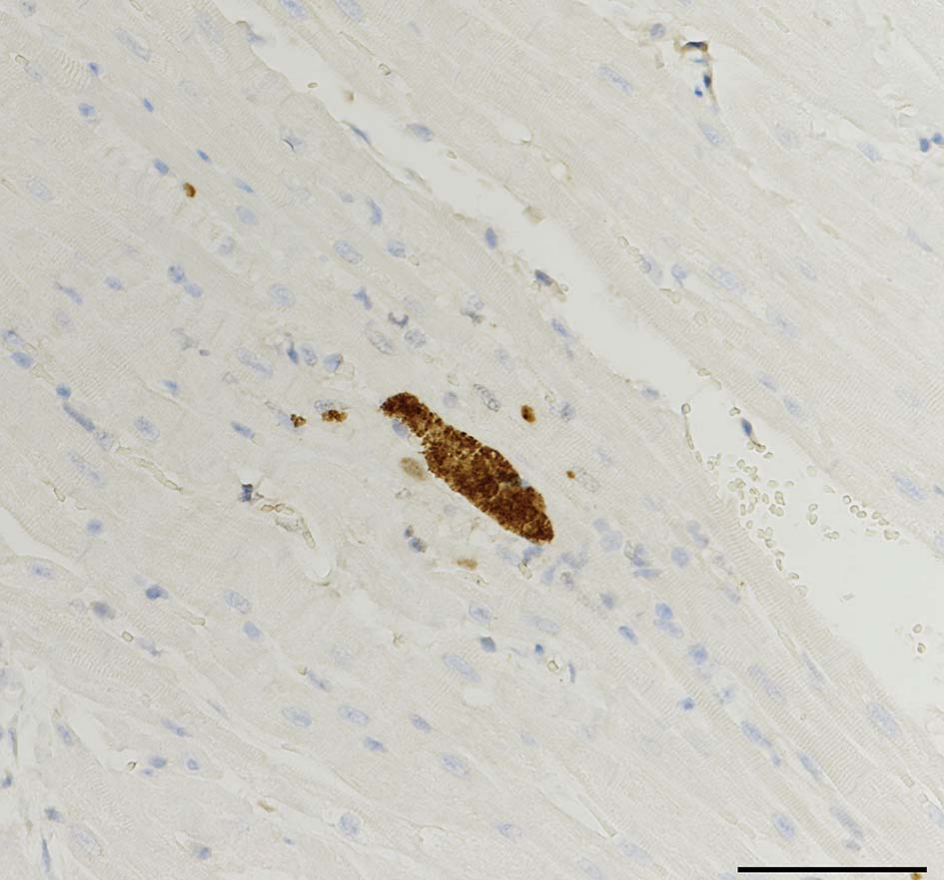
Cyst-like structure potentially attributable to a coccidian parasite observed in heart tissue of a Galapagos sea lion pup (ID 18). Bar = 100 μm.

Cross sections were made from pieces of heart and brain taken from 26 of the 29 autopsied corpses. On histological examination under a microscope, only one slide (individual 18) revealed the presence of a cyst-like structure potentially attributable to coccidian parasites (Fig. 2). However, the heart, brain and lung samples from this individual were negative on PCR, and the heart and brain samples came back negative in the immunohistochemistry test.

### Molecular detection

*Toxoplasma gondii* DNA was explored in 29/29 individuals sampled in 2021 (heart, brain, and lung sections). It was detected only in a lung sample of individual 17 (in duplicate with a Cq mean of 39.38).

## Discussion

Results from serological analyses demonstrate the frequent exposure of Galapagos sea lions sampled in 2016–2017 and in 2021 to *T. gondii*. This represents a significant finding given the potential health risks this parasite poses to naïve sea lions, already vulnerable due to their limited range and population size [36]. Furthermore, the detection of *T. gondii* DNA in one individual points to the possibility of health issues related to *T. gondii* infection, supporting the need for continued monitoring and research to understand its impact on sea lion health.

The 79% seroprevalence in wild-sampled juvenile Galapagos sea lions (2016–2017 sample) falls within the upper range of seroprevalence reported for marine mammals, such as 76.9% in Southern elephant seals (*Mirounga leonina*) from Antarctica [35] and 61.1% in California sea lions (*Zalophus californianus*) from California [2]. However, lower prevalence values have been documented in other Antarctic species such as Crabeater seals (*Lobodon carcinophaga,* 50%) and Weddell seals (*Leptonychotes weddellii*, 41.9%) [35], California sea lions (29.6%) from Alaska [2], Ringed seals (*Pusa hispida*, 26%) from Canada [33], and New Zealand sea lions (6%) [25]. These variations could be related to differences in diet, habitat, and geographic proximity to felid populations responsible for contaminating the ecosystems with *T. gondii* oocysts [22].

A lower seroprevalence (21%) was found in carcasses of sampled Galapagos sea lions in 2021. While this could be related to a low sample size, age class could also play an important role, as seen for the California sea lion [2]. In this species, the occurrence of *T. gondii* infection in aborted fetuses highlights vertical transmission, while increasing exposure with age is consistent with additional opportunities for horizontal transmission over time [2]. An increasing prevalence of antibodies against *T. gondii* with age due to prolonged environmental exposure and bioaccumulation of the parasite through the food chain are observed in marine mammal species [1, 2]. Therefore, the low prevalence in the 2021 samples of Galapagos sea lions could be due to the predominance of pups in the sampled population, which are less likely to have been exposed to the parasite compared to juveniles and adults.

Detection of *T. gondii* DNA in a Galapagos sea lion less than two years of age (No. 17) that was also positive for antibodies against *T. gondii* raises questions about the possible infection routes in this age class. Unlike most pinnipeds, Galapagos sea lions feed exclusively on maternal milk during their first year and gradually begin to feed themselves during their second year [46, 48]. Thus, this individual could not have been exposed through consumption of fish carrying oocysts, suggesting that congenital transmission is possible, as documented in California sea lions [2]. However, exposure of pups to contaminated soil cannot be excluded since it is common to detect cat feces in areas near Puerto Baquerizo Moreno [3]. In the El Malecón, dogs and cats are broadly present including at the rookeries, which would increase the probability of exposure to various parasites in young sea lions [4], since they remain permanently on their birth beach until they are over 6 weeks of age and only start swimming around 2–3 months of age, always staying close to the beach [46].

Histopathology analyses yielded inconclusive results, with only one individual (No. 18) showing a tissue cyst-like structure in the heart, but without confirmation by immunochemistry. It is worth noting that the constraints inherent to sample collection in the remote Galapagos archipelago, storage space limitations and need to reduce sample transport costs, did not allowed us to collect the entire organs of the autopsied sea lions but only tissue samples from regions identified as priorities for molecular biology analyses, such as the apex of the heart [9]. By proceeding this way, we may not always have sampled tissue parts likely to harbor *T. gondii* tissue cysts. These are generally found predominantly in the cardiac muscle, but their distribution among and within organs can be highly variable [9]. It is also possible that the parasitic load of the autopsied sea lions allowed for *T. gondii* detection *via* molecular biology without being sufficient for cysts to be observed in histopathology, as sometimes reported for sea otters [26].

However, there are some limitations of this study that must be considered when interpreting the results. First, the lack of adults in our study could underestimate the overall prevalence within the population. An expanded serological survey to several age groups in Galapagos sea lion colonies is needed to confirm the hypothesis of predominantly age-associated *T. gondii* exposure in this species. Second, further research on the sources of exposure and transmission dynamics of this parasite in their population is needed, given the various possible exposure routes to *T. gondii*. Third, future efforts should aim to collect and analyze complete organs to better assess the presence of tissue cysts and associated lesions. This approach would provide a more reliable estimate of the pathogenicity of *T. gondii* in Galapagos sea lions. Finally, even though the modified agglutination test (MAT) that we used is the most common serological test for marine mammals and is practical for field studies due to its simplicity and low cost, its sensitivity for Galapagos sea lions should be determined and compared to other methods like the indirect fluorescence antibody test (IFAT), which may lead to underreporting of seropositive individuals [1, 12, 44]. This preliminary validation step needs to be conducted in Galapagos sea lions. In this sense, although it is undeniable that our results show exposure of Galapagos sea lions to *T. gondii*, the evidence that this is a threat to the viability of populations is controversial. Therefore, we suggest applying the precautionary principle which requires actions to protect biodiversity when there is a plausible risk, such as the control of feral cats, while definitive studies are conducted. It is essential to include consultation with all stakeholders to implement precautionary measures that are widely supported and are applicable to local circumstances [7].

## Conclusion

This study provides, for the first time, evidence of exposure to *T. gondii* of Galapagos sea lion juveniles sampled in 2016–2017 and pups of this species sampled in 2021 by serological analyses. In addition, the detection of *T. gondii* DNA in one individual represents a significant finding, given the potential health risks this parasite poses to endangered Galapagos sea lions. Continuous monitoring and further research are needed to improve our understanding of the impact of *T. gondii* on Galapagos sea lion health and transmission dynamics, and to implement appropriate conservation strategies.
